# Eukaryotic Cells Producing Ribosomes Deficient in Rpl1 Are Hypersensitive to Defects in the Ubiquitin-Proteasome System

**DOI:** 10.1371/journal.pone.0023579

**Published:** 2011-08-12

**Authors:** Kerri B. McIntosh, Arpita Bhattacharya, Ian M. Willis, Jonathan R. Warner

**Affiliations:** 1 Department of Cell Biology, Albert Einstein College of Medicine, Bronx, New York, United States of America; 2 Department of Biochemistry, Albert Einstein College of Medicine, Bronx, New York, United States of America; University of Louisville, United States of America

## Abstract

It has recently become clear that the misassembly of ribosomes in eukaryotic cells can have deleterious effects that go far beyond a simple shortage of ribosomes. In this work we find that cells deficient in ribosomal protein L1 (Rpl1; Rpl10a in mammals) produce ribosomes lacking Rpl1 that are exported to the cytoplasm and that can be incorporated into polyribosomes. The presence of such defective ribosomes leads to slow growth and appears to render the cells hypersensitive to lesions in the ubiquitin-proteasome system. Several genes that were reasonable candidates for degradation of 60S subunits lacking Rpl1 fail to do so, suggesting that key players in the surveillance of ribosomal subunits remain to be found. Interestingly, in spite of rendering the cells hypersensitive to the proteasome inhibitor MG132, shortage of Rpl1 partially suppresses the stress-invoked temporary repression of ribosome synthesis caused by MG132.

## Introduction

The synthesis of ribosomes is a special challenge to the cell in two respects. First, in a growing cell it utilizes a very substantial fraction of the cell's resources [1]. Perhaps more importantly, it requires the coordinated production of each of the 79 ribosomal proteins (RPs), which are needed, with a couple of exceptions, in exactly equimolar amounts. There is substantial evidence from both yeast and metazoan systems that an imbalance of RPs can lead to stress, now often termed ‘nucleolar stress’ (reviewed in [2],[3]). Thus, in yeast overproduction of a RP from a 2 micron plasmid drives the plasmid number down several fold [4]. In *Drosophila* haploinsufficiency for a RP leads to the *minute* phenotype, with delayed development, short bristles, etc. [5]. In zebrafish haploinsufficiency for any of at least 14 RPs leads to tumor formation [6]. In mammalian cells haploinsufficiency or excess amounts of a RP lead to accumulation of p53 and subsequent cell cycle arrest or apoptosis [7], [8]. In humans haploinsufficiency for any of several RPs leads to Diamond-Blackfan anemia and associated pathology, including increased incidence of cancer [9]. Other examples of pathological effects of haploinsufficiency for RP genes are appearing with increasing frequency (Reviewed in [10], [11]). Without doubt, the role of ribosomopathies in human disease is only just beginning to be appreciated.

While the yeast *Saccharomyces cerevisiae* is missing certain of the components that lead to such pathology, e.g., p53, it nevertheless is an attractive model to probe some of the more general responses to nucleolar stress. Under the assumption that deficiency of a single RP would lead to substantial amounts of incomplete ribosomal subunits that should be subject to surveillance and degradation, we carried out Synthetic Genetic Array analyses [12] using strains constructed with deletions of one of the two paralogues of a RP gene, *RPL1B*, *RPL4A*, or *RPS6A*, each crossed into the *S. cerevisiae* knock-out collection. The summary results have been reported previously [13]. We now concentrate on the effects of deletion of *RPL1B*, which has some unusual characteristics of its own.

Surprisingly, although Rpl1 is essential for growth, 60S subunits can be assembled in the absence of Rpl1, and these can be incorporated into polyribosomes. Nevertheless, cells limited for Rpl1 grow slowly, are synthetically sick with deletion of numerous members of the ubiquitin-proteasome system, and are hypersensitive to the proteasome inhibitor MG132.

## Results

### Pseudo-haploidy for RP genes

The original aim of these experiments was to assess how the cell responds to a shortage of a single RP. In *S. cerevisiae* this is experimentally simple to arrange, since many RPs are encoded by two genes that in most cases yield identical or nearly identical proteins. We selected three RPs for which to assay the effects of a deficiency on rate of growth and on genetic interactions: Rpl1 (Rpl1 in *E. coli*, Rpl10a in mammals), Rpl4 (Rpl4 in *E. coli* and mammals) and Rps6 (Rps6 in mammals, not present in *E. coli*). Rpl1 and Rpl4 are both essential, universally conserved RPs whose placement in the 60S subunit is well-defined in both bacteria and eukaryotes [14], [15]. Rpl4 is known to facilitate early assembly of the 60S subunit and to make extensive contact with multiple domains of 25S rRNA, including Domain I, the first to be transcribed, suggesting an obligatory early assembly into the 60S subunit [14]. In contrast, Rpl1 occupies a peripheral location in the 60S subunit with only localized rRNA contacts. Nevertheless, it forms a major structural feature of the ribosome, the L1 arm, which plays an important role in removal of E-site tRNA [16]–[18]. Thus, limiting for Rpl1 and Rpl4 would perturb very different stages of subunit assembly. Rps6 was included for comparison as a component of the 40S subunit known to be essential for subunit assembly [19], [20]. For each of the three RPs the paralogous genes yield identical proteins.

**Figure 1:**
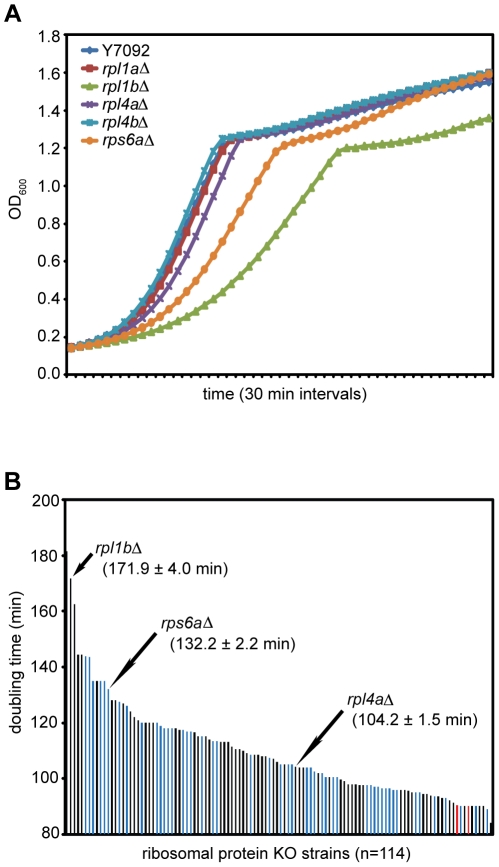
Growth of strains carrying deletions of one RP gene paralogue. (A) Growth curves for wildtype (Y7092) and RP KO strains in YPD at 30°C. OD_600_ was read every 30 min in a Bioscreen C™ microbiology reader. Lag phases have been excluded from each curve to show growth from OD_600_ ∼0.1. (B) Doubling times for paralogous RP KOs, calculated using Bioscreen growth curves (see Materials & Methods). Doubling times for 60S RPs are shown in black, 40S RPs are in blue, and wildtype (Y7092 and BY4741) are in red. Arrows indicate positions of *rpl1b*Δ, *rpl4a*Δ, and *rps6a*Δ, the nat^r^-marked KO strains (Y7092 background) used in this study. Doubling times given are mean value ± SE (*rpl1b*Δ, n = 13 independent biological replicates; *rpl4a*Δ, n = 8; *rps6a*Δ, n = 6). All other RP KO strains were from the Open Biosystems kanMX-marked KO library (BY4741 background); values represent the mean of three technical replicates.

Deletion of one of the genes encoding each of these RPs, a pseudo-haploinsufficiency, leads to slower growth, but over a wide range, with deletion of *RPL1B* having the most deleterious effect (Fig. 1A). This may be due partially to an imbalance between the two genes encoding Rpl1. qPCR analysis reveals that deletion of *RPL1B* reduces the Rpl1 mRNA by 55%, consistent with the published value [21]. To put the slow growth of the *rpl1b*Δ strain in context, we determined the growth rate of strains carrying deletions (KOs) of most of the individual RP paralogous genes of yeast (Fig. 1B). Deletion of *RPL1B* had a greater effect on growth than all but one of the RP paralogues. Nevertheless, normal growth could be restored by overexpression of *RPL1A*, showing that the *rpl1b*Δ growth defect is not due to a paralogue-specific function. The reasons for the spread of growth rates in this set of pseudo-haploids are not clear but could be due to relative abundance of transcripts between the paralogue pairs, or to some RPs being normally made in excess, as appears to be the case in mammalian cells [22], or, more interestingly, through compensation by the remaining gene, as in the case of Rps14 [23].

### RPL1 is dispensable for 60S subunit assembly in yeast

**Figure 2:**
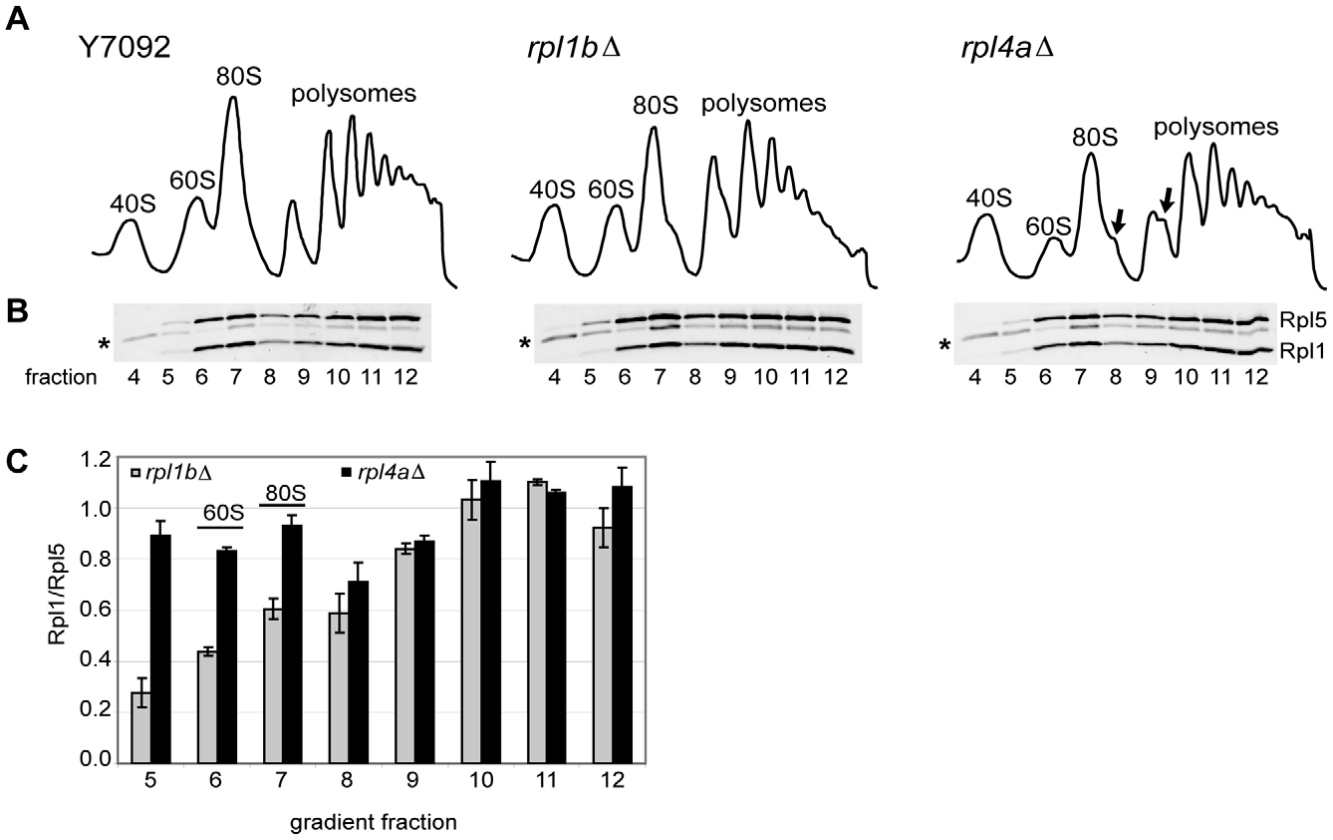
60S subunits can be made without Rpl1. (A) Polysome profiles of whole cell lysates of the indicated strains (See Methods). 8–10 A_260_ units of whole cell lysate were centrifuged for 2.5 h at 40K rpm on a 10–50% sucrose gradient. Top of the gradient is at left; arrows indicate half-mers. (B) Western blot analysis of equal volumes of each gradient fraction, shown below corresponding polysome profiles. α-Rpl5 cross-reacts with a 40S protein (middle band marked with an asterisk, under 40S, 80S, and polysome peaks). (C) The Rpl1/Rpl5 ratio was determined for each fraction of the gradients. The values for *rpl1b*Δ and *rpl4a*Δ in are shown, normalized to the ratio for Y7092, ± SD (n = 2).

The very slow growth of *rpl1b*Δ compared to other RP KOs led us to re-examine the molecular consequences of Rpl1 limitation. Polysome profiles indicated that a reduction in Rpl1 has somewhat different consequences for ribosome synthesis than expected from the loss of a 60S RP (Fig. 2A). A typical polysome profile of cells with impaired 60S synthesis is illustrated by the strain carrying *rpl4a*Δ: the level of free 60S subunits is reduced, and half-mer polysomes indicate an excess of 43S initiation complexes stalled on mRNA, waiting for 60S subunits [24]. In contrast, the strain carrying *rpl1b*Δ shows little reduction of the 60S subunit peak and few half-mer polysomes. There is, however, a substantial reduction in the levels of the largest polysomes, suggesting a limitation in the initiation process.

A possible explanation for the only slight reduction in free 60S subunits and the few half-mers seen in the *rpl1b*Δ polysome profile is that 60S synthesis continues even in the absence of Rpl1, resulting in structurally or functionally defective subunits. Using Rpl5 as a control, Western blot analysis of the relative amount of Rpl1 in *rpl1b*Δ 60S subunits and ribosomes showed, indeed, that the ratio of Rpl1 to Rpl5 in the 60S fractions of *rpl1b*Δ was only about one third that of wildtype (Fig. 2B,C). By contrast, in the *rpl4aΔ* strain the Rpl1/Rpl5 ratio of the few 60S subunits was normal. This comparison supports the hypothesis that whereas no 60S subunit can be made lacking Rpl4, subunits can be assembled without Rpl1.

**Figure 3:**
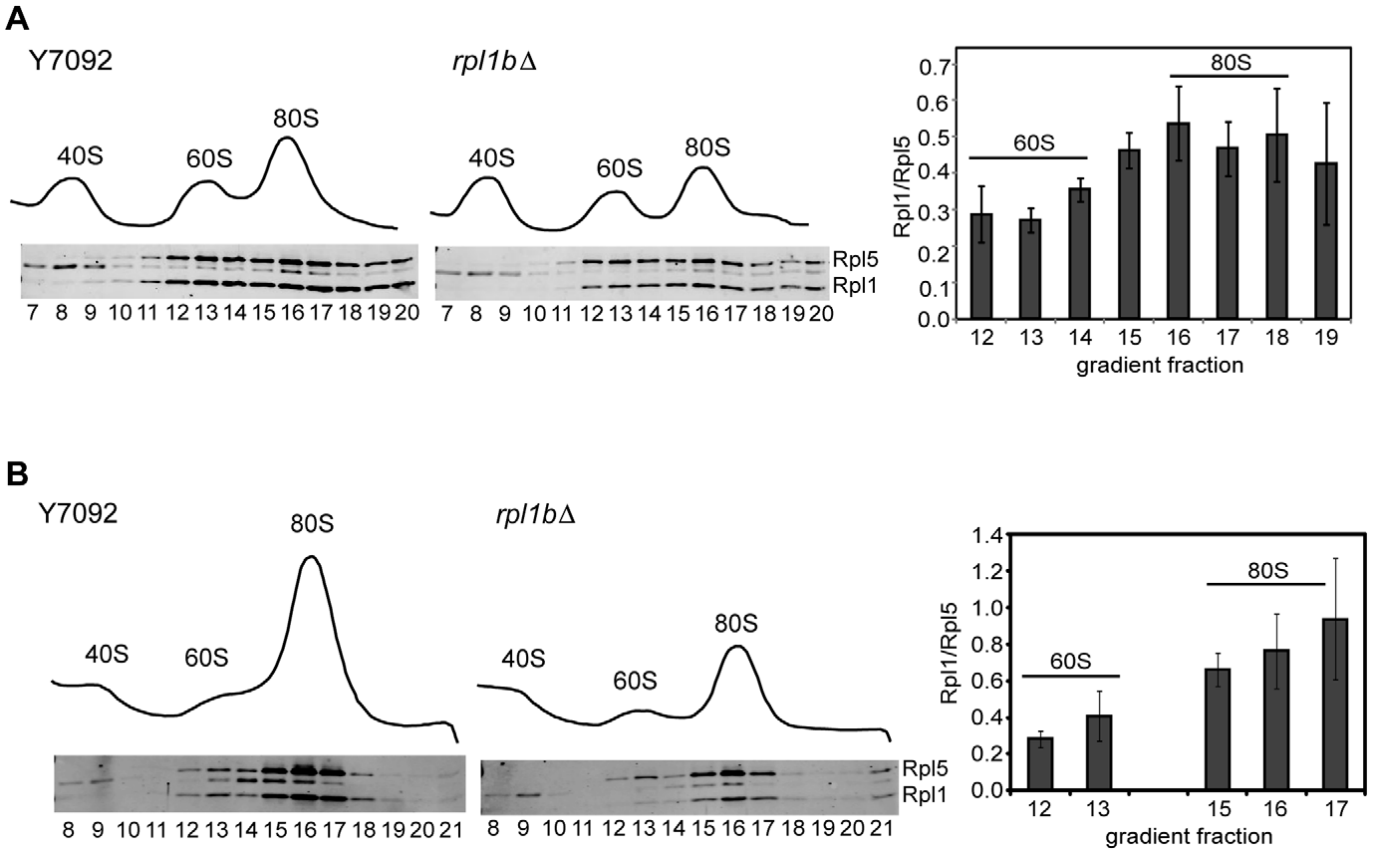
Subunits without Rpl1 can be exported to the cytoplasm. (A) Polysome profiles of whole cell lysate; in this case 5–20% gradients were used to better separate 60S from 80S peaks. As above, 8–10 A_260_ units of lysate was layered onto sucrose gradients and centrifuged for 3 h at 40K rpm. Western blots of gradient fractions are shown below the polysome profiles; Rpl1/Rpl5 ratio for *rpl1b*Δ in each fraction is graphed normalized to the wildtype ratio, ± SE (n = 3). (B) Polysome profiles of cytoplasmic extracts: As above, except using approximately 3 A_260_ units of cytoplasmic extract, prepared as described in Methods. The freedom from nuclear contamination was demonstrated by western blot analysis (Fig. S1). Only peak fractions with enough band intensity for accurate quantification are graphed (± SD, n = 2).

Interestingly, although Rpl1 was reduced in 60S subunits, the Rpl1/Rpl5 ratio was at nearly wildtype levels in the *rpl1b*Δ polysome fractions, indicating a possible selection against the Rpl1-deficient subunits in actively translating ribosomes. Analysis on 5–20% sucrose gradients, in order to better resolve 60S from 80S particles, showed that although the greatest reduction in Rpl1 is seen in 60S subunits (to ∼30% of wildtype levels), *rpl1b*Δ also shows a distinct reduction of Rpl1 in 80S monomers (to ∼50% of wildtype levels) (Fig. 3A). This suggests that at least a fraction of subunits lacking Rpl1 can indeed join with 40S subunits, although not necessarily engage in translation.

### Subunits lacking Rpl1 are competent for export and assembly into ribosomes

In many cases aberrant or incomplete 60S ribosomal subunits are confined to the nucleus, their export being blocked [25], [26]. In order to establish that the subunits lacking Rpl1 are actually exported from the nucleus, we analyzed cytoplasmic extracts from wildtype and *rpl1b*Δ cells (Fig. 3B, Fig. S1). Western blots of the cytoplasmic extract fractions confirmed that the Rpl1/Rpl5 ratio in 60S subunits was only ∼30% of wild type and that of 80S ribosomes was only ∼60% of wildtype levels. Thus, 60S subunits lacking Rpl1 are present in the cytoplasm and potentially available for initiation of translation.

### Subunits lacking L1 can be found in polysomes

**Figure 4:**
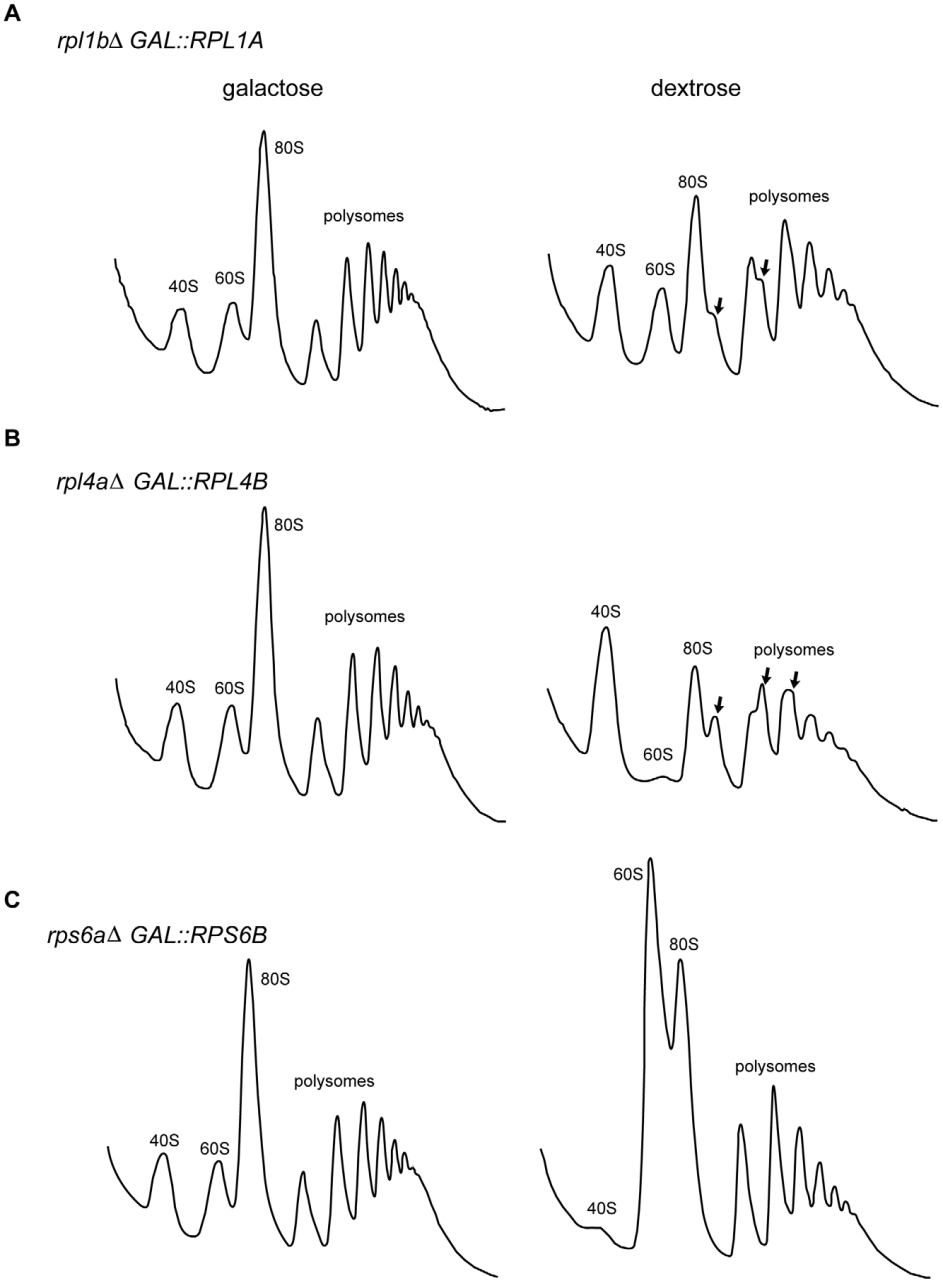
Comparison of polysome profiles after depletion of (A) Rpl1 (B) Rpl4 and (C) Rps6. Strains carrying a knockout of one RP paralogue with the other paralogue under control of the *GAL1* promoter were grown to early log phase in medium supplemented with 2% galactose. Cultures were then filtered, the cells washed and split into YP supplemented with either 2% galactose or 2% dextrose. After 2 h, the cultures were harvested and analyzed as in Fig. 2. Arrows indicate half-mer polysomes.

For a more thorough analysis of the synthesis of 60S ribosomal subunits in the absence of Rpl1, we developed a strain in which the synthesis of Rpl1 could be completely repressed (*rpl1b*Δ, *GAL1::RPL1A*) along with control strains to repress synthesis of Rpl4 (*rpl4a*Δ, *GAL1::RPL4B*) or Rps6 (*rps6a*Δ, *GAL1::RPS6B*). Due to the rapid turnover of RP mRNAs, levels of *RPL1* and *RPS6* transcripts are reduced by 98–99%, and *RPL4* mRNA by >90%, after 60 minutes in dextrose medium, as determined by qPCR. Polysome profiles of these strains after two hours in dextrose show that in cells deprived of Rpl4 there are essentially no free 60S subunits, and the half-mer peaks actually exceed the normal ones (Fig. 4B). Depletion of Rps6 leads to an enormous peak of free 60S subunits (Fig. 4C). In both cases a substantial imbalance between the two subunits has developed. By contrast, depletion of Rpl1 has relatively little effect on the pattern (Fig. 4A).

**Figure 5:**
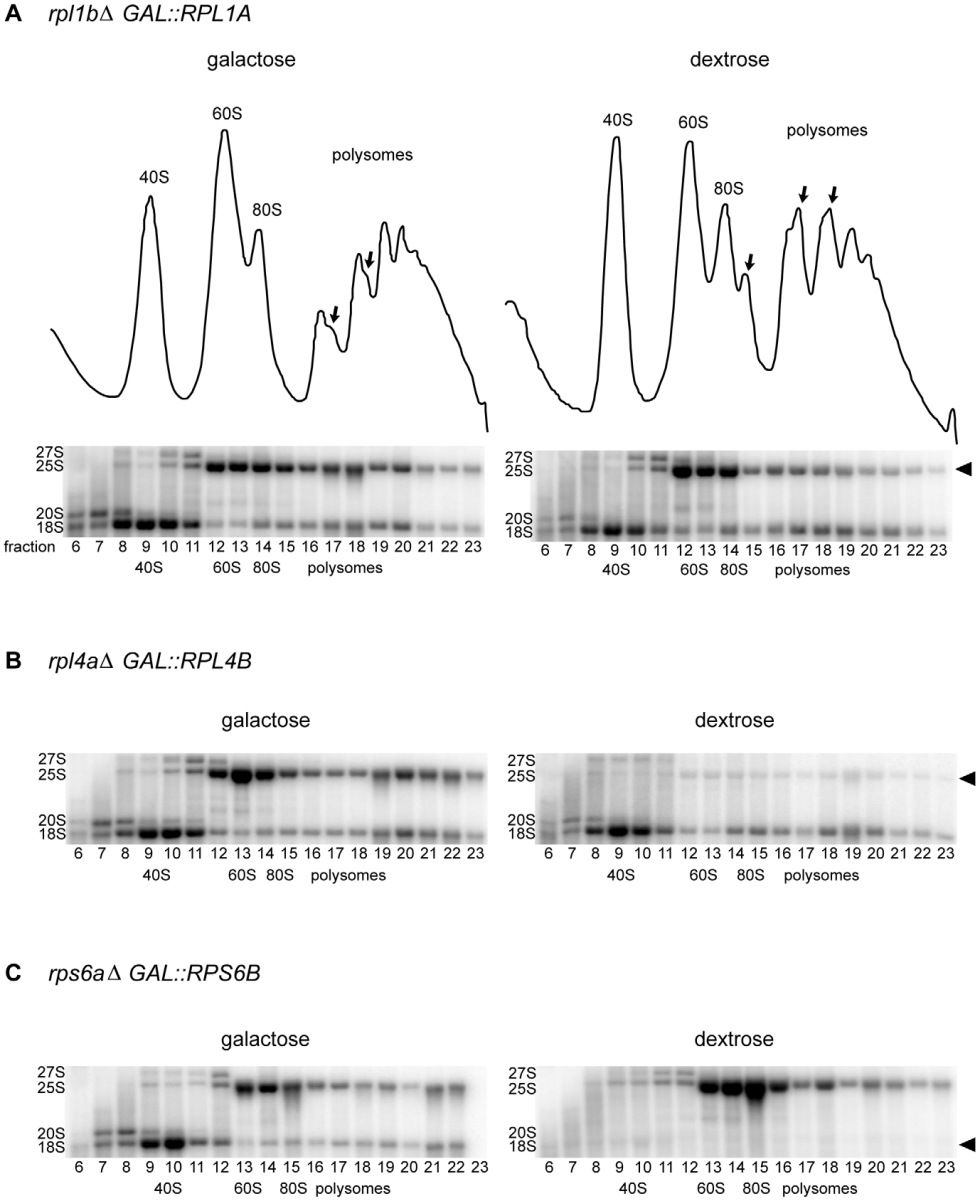
Subunits made without Rpl1 can be incorporated into polysomes. Rpl1 (A) Rpl4 (B) and Rps6 (C) repressible strains were grown to log phase in phosphate-depleted medium (see Methods) supplemented with 2% galactose, filtered, and transferred to phosphate-depleted medium supplemented with either 2% galactose or 2% dextrose. After 60 min, 100 µCi/ml ^32^P was added to each culture. After a further 60 min, cells were harvested and total lysate was analyzed as in Fig. 2, except that both lysing buffer and gradient buffer contained Mg^2+^ at 1.5 mM to separate subunits not involved in translation (see Methods). Polysome profiles for the Rpl1 depletion strain in galactose or dextrose media are shown in (A), half-mers are indicated by arrows. RNA from equal volumes of each fraction was analyzed on a denaturing gel, blotted, and subjected to autoradiography (see Methods). rRNA corresponding to the subunit of the depleted RP for each strain is indicated by arrowheads flanking the blots for strains grown in dextrose.

This result further supports the conclusion that synthesis of 60S subunits continues even in the absence of Rpl1. As a more definitive test, we labeled the repressible strains for 60 minutes with ^32^P orthophosphate, either during growth in galactose or between 60 and 120 minutes following transfer to dextrose. For these polysome gradients we employed 1.5 mM Mg^2+^, which more closely replicates physiological Mg^2+^ levels [27], and which dissociates subunits not translating mRNA [13]. It is interesting that the proportion of half-mers increases substantially under these conditions of analysis (Compare Fig. 5A with Fig. 4A), suggesting that some 60S subunits, presumably those lacking Rpl1, are loosely associated with polysomes.

Autoradiographs of samples from the gradients (Fig. 5A–C) show clearly that while lack of Rps6 leads to synthesis of 60S but not 40S subunits, and lack of Rpl4 leads to synthesis of 40S but not 60S subunits, lack of Rpl1 permits substantial synthesis of both 60S and 40S subunits. Analysis of total RNA on a separate gel showed that repressing conditions reduced 25S rRNA synthesis by ∼35% for the *rpl1b*Δ strain, but more than 95% for the *rpl4a*Δ strain. Similarly, 18S rRNA synthesis was reduced by more than 90% for the *rps6a*Δ strain. Furthermore, many of the 60S subunits synthesized after depletion of Rpl1 are associated with polysomes. However, the ratio of the radiolabelled 25S rRNA to 18S rRNA is substantially less in the polysomes than in the 80S or the subunit peaks (Fig. 5A), showing that while 60S subunits lacking Rpl1 can associate with polysomes, they are strongly discriminated against during translation initiation.

The apparent conflict between Fig. 2C, where we did not detect Rpl1-deficient 60S subunuits on polysomes, and Fig. 5A, where we did, is likely due to both physiological and experimental effects. We suggest that in the *rpl1b*Δ strain there is a competition between complete and Rpl1-deficient 60S subunits that greatly favors the complete ones. Only when a large proportion of Rpl1-deficient 60S subunitsaccumulates, after two hours in dextrose medium, can they overcome the competition to force their way onto polysomes. Experimentally, the Rpl1/Rpl5 signal to noise ratio in western blots is inadequate to detect in polysomes the small fraction of defective 60S subunits that may be present in the Δ*rpl1b* strain. Indeed, in polysomes of cells entirely depleted of Rpl1 for two hours, the Rpl1/Rpl5 ratio in the polysomes declined to ∼0.8 (data not shown?).

An interesting sidelight to Fig. 5 is the observation that in these low Mg^2+^ conditions the precursor particles containing either 27S or 20S pre-rRNA migrate more slowly than the mature subunits. Although they have greater mass, with numerous extra proteins [28], the precursor particles must be substantially less compact at this low Mg^2+^ concentration, providing more hydrodynamic resistance to sedimentation.

### Partial turnover of Rpl1-deficient 60 S subunits

**Figure 6:**
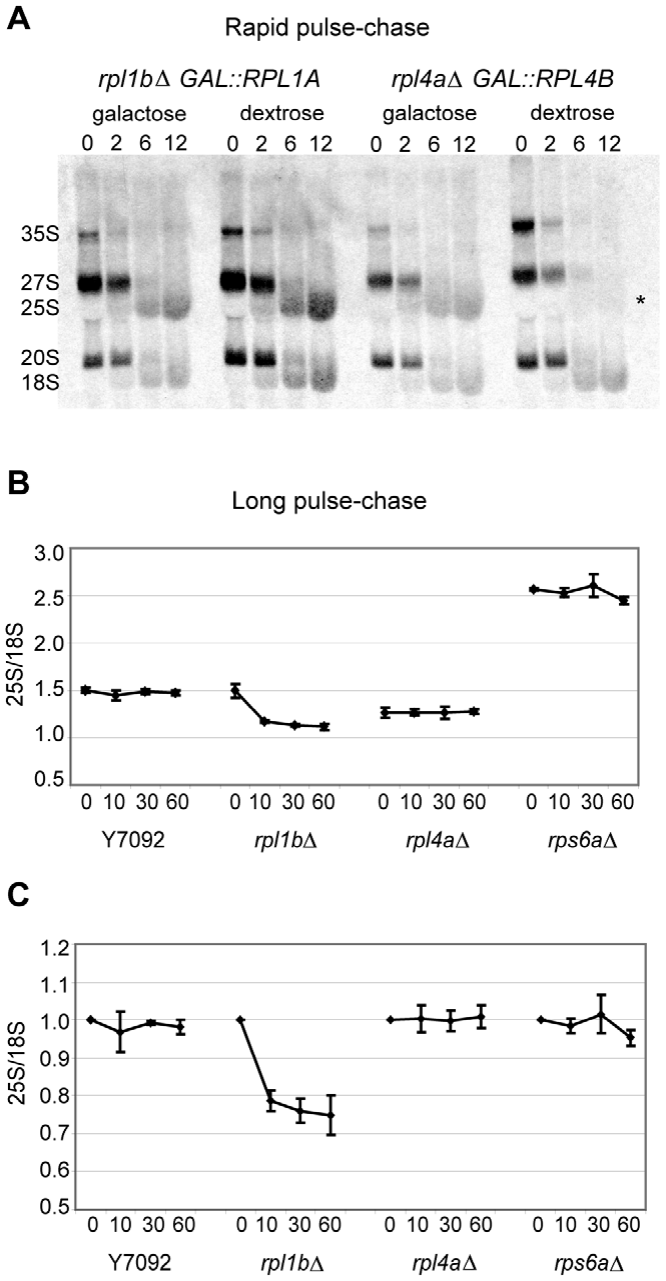
A fraction of subunits lacking Rpl1 are targeted for degradation. (A) Rapid pulse-chase. Cultures of the indicated genotype were labeled with a three minute pulse of [C^3^H_3_]-methionine, with a subsequent chase with unlabelled methionine, as described in Methods, either in galactose-containing medium, or after 30 minutes in dextrose-containing medium. Total RNA (5 µg per sample) was run on a 1.5% denaturing agarose gel, blotted, then subjected to autoradiography. (B)Long pulse-chase – In this experiment cultures of the indicated genotype were labeled for 15 min with [C^3^H_3_]-methionine, followed by a cold chase. By 15 minutes most of the label is in mature 25S and 18S species. RNA was analyzed as above. In addition, the identified 25S and 18S bands were cut from the membrane and their CPM of ^3^H determined in a scintillation counter. The data is presented to show the 25S CPM/18SCPM ratio (± SE; n = 4), highlighting the relative amounts of rRNA species in RP mutants relative to wildtype. (C) The data from (B), normalized to show decay from t = 0.

Note that in gradient fractions of neither the Rpl4-depleted nor the Rps6-depleted cells can the corresponding 27S or 20S pre-rRNA be detected (Fig. 5B, C), suggesting that the nascent subunits missing key proteins are rapidly degraded. We confirmed this rapid degradation with a pulse-chase experiment where a short pulse of [C^3^H_3_]-methionine, which labels the numerous methyl groups on rRNA, was followed by a short chase. After a 30 minute depletion of Rpl4, newly synthesized 27S rRNA is degraded within <six minutes (Fig. 6A). In contrast, Rpl1 depletion does not affect the processing of 27S precursor; mature 25S rRNA is produced.

Since the subunits lacking Rpl1 appear to be selected against in polysomes, we hypothesized that these subunits might be degraded. This was tested with a somewhat different pulse-chase (Fig. 6B, C). Strains bearing *rpl1bΔ*, *rpl4aΔ*, or *rps6aΔ* were grown in supplemented minimal medium, pulsed with [C^3^H_3_]-methionine for 15 minutes and then chased with an excess of unlabelled methionine. The resulting 25S/18S ratio is shown in Fig. 6B, C. A 15 minute pulse is sufficiently long that the 25S/18S ratio of wildtype cells remains constant during the chase. As expected, cells missing *RPS6A* are substantially deficient in 18S rRNA, while cells lacking *RPL4A* are slightly deficient in 25SrRNA (Fig. 6B). In both cases the ratio has been established by the end of the pulse and does not change. By contrast, cells deficient for Rpl1 have approximately wildtype levels of 25S rRNA at time zero, but show a turnover of ∼20–25% of the 25S rRNA within the first ten minutes (Fig. 6B, C). The remainder remains stable. This result suggests that unlike cells deficient in Rpl4 or Rps6, in which the pre-rRNA molecules are rapidly degraded (Fig. 6A), a substantial fraction of the pre-60S subunits lacking Rpl1 are stable, while only some are degraded.

### Genetic interactions of RP-deficient strains

Under the assumption that deficiency of a RP would lead to substantial amounts of incomplete ribosomal subunits that should be subject to surveillance and degradation, we carried out Synthetic Genetic Array analyses [12] by mating *rpl1b*Δ, *rpl4a*Δ, and *rps6a*Δ with the *S. cerevisiae* KO collection. The overall results have been reported previously [13]. We now concentrate on the striking contrast between the collection of genes that interact with Δ*rpl1b* compared to the other RP KOs. In brief, some 15 genes of the ubiquitin-proteasome, SUMO, and urmylation pathways were synthetic sick with *rpl1b*Δ [*CUE3*, *DOA1*, *PRE9*, *RPN4*, *RPN10*, *RUP1*, *SEM1*, *UBA4*, *UBC4*, *UBI4*, *UBP2*, *UBP6*, *UBP8*, *ULS1*, *URM1*], while none were with *rpl4a*Δ and only three with *rps6a*Δ. The breadth of this list of interacting genes is so broad is presumably due to an active demand for protein turnover, but is it turnover of the defective ribosomal subunits themselves, or turnover of defective proteins made by the ribosomes lacking Rpl1?

We carried out a number of experiments to try to detect aberrant proteins or their degradation. Western blots of whole cell lysates probed with anti-ubiquitin showed at best a minor increase in polyubiquitin conjugates in *rpl1b*Δ compared to wildtype or *rps6a*Δ (data not shown). A [C^3^H_3_]-methionine pulse-chase yielded no detectable differences in bulk protein turnover between *rpl1b*Δ and wildtype strains (data not shown). Since it has been suggested that Rpl1 influences the removal of empty tRNA from the E site of the ribosome, and that the removal of the E site tRNA is coupled to the accurate filling of the A site [29], we hypothesized that lack of Rpl1 might make the ribosome more susceptible to frame-shifting or to miscoding. However, constructs designed to detect frame-shifting [30], [31] or miscoding [32] yielded no detectable effects in the *rpl1b*Δ strain.

**Figure 7:**
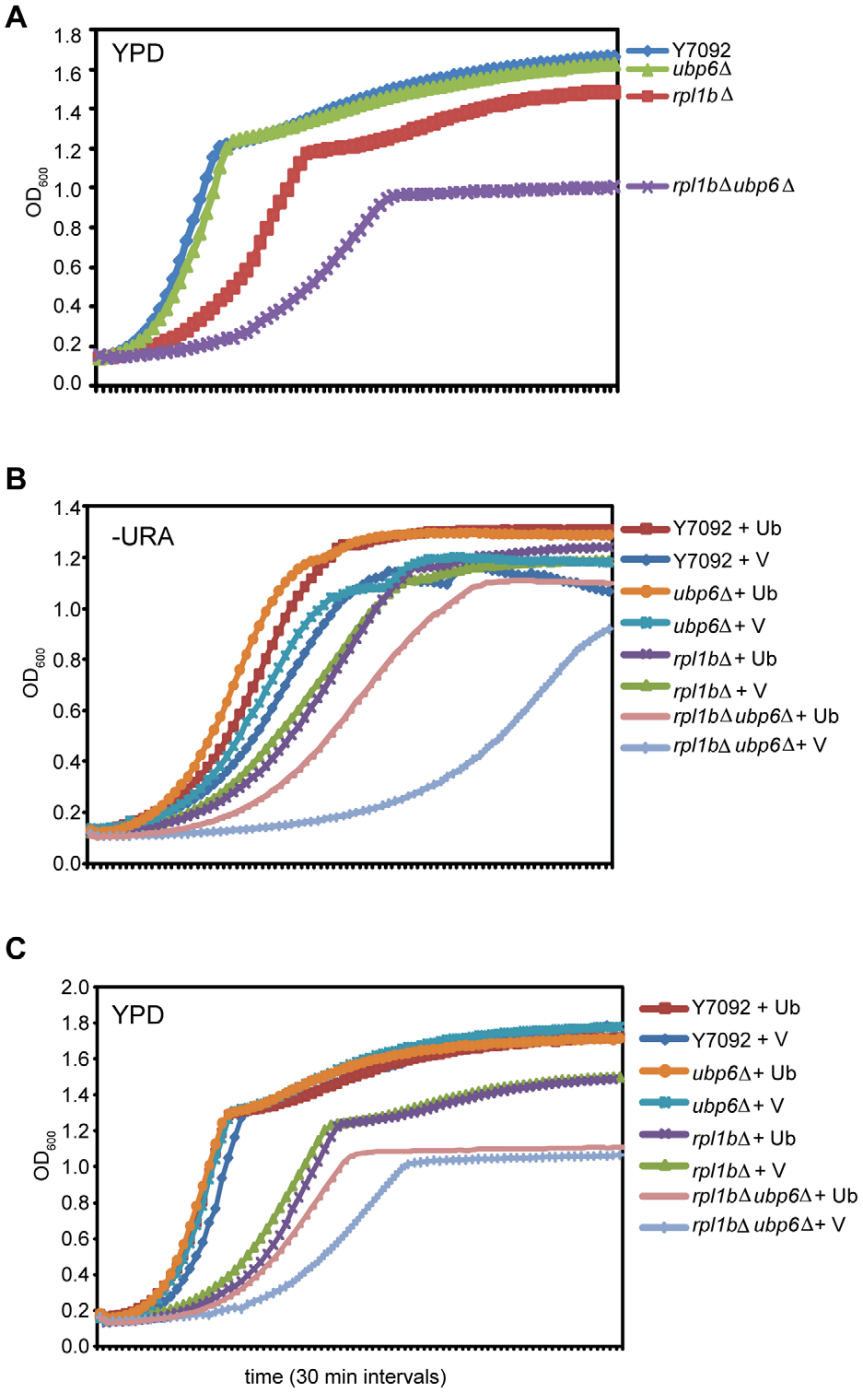
Effects of *Δubp6* on strains lacking *RPL1B*. (A) Growth curves of the indicated strains in YPD. (B) Growth curves in minimal medium without uracil of the indicated strains carrying either the 2 micron vector pRS426 (V) or the 2 micron plasmid pUB175 carrying ubiquitin (Ub). (C) As in (B) in YPD medium.

Having failed to detect any increase in defective translation products or their turnover in cells carrying *rpl1b*Δ, we examined the possibility that proteins derived from the genes identified in the SGA screen were involved in the turnover of Rpl1-deficient 60S subunits. Two of the slowest growing but still viable combinations, *ubp6*Δ *rpl1b*Δ, and *doa1*Δ *rpl1b*Δ were freshly constructed for further experiments. Ubp6 deubiquitinates polyubiquitinated substrates before they enter the proteasome for degradation, freeing the ubiquitin moieties for re-use [33]. The slow growth of the *ubp6*Δ *rpl1b*Δ strain (T_1/2_ = 250±5 min.) is apparent in Fig. 7A. Tables S3 and S4 provide some of the characteristics of this strain. It has less RNA/OD_600_, suggesting fewer ribosomes per cell. This is substantiated by a two-fold lower level of mature 25S and 18S rRNA, suggesting that ribosome synthesis is somewhat repressed. Northern analysis showed substantially less 35S, 27S, and 20S pre-rRNA species, corroborating this conclusion. The polysome gradient had fewer ribosomes, but the pattern resembled the wt, with more large polysomes (Fig. S2A). Surprisingly, the double mutant no longer has a deficit of Rpl1 in the 60S subunits; the Rpl1/Rpl5 ratio in the 60S subunits is similar to that on polysomes (Fig. S2A). This result suggests that Ubp6 itself is not involved with the degradation of 60S subunits lacking Rpl1, but that its absence leads to repression of ribosome synthesis for which the limited supply of Rpl1 is sufficient. The growth curves in rich medium have two components, a log phase of rapid growth by fermentation of glucose to OD_600_ ∼1.2, followed by a diauxic shift to oxidative metabolism. The strain carrying the double mutant *ubp6*Δ *rpl1b*Δ cannot carry out the latter, nor can it grow on non-fermentable medium (not shown) although both single mutants can (Fig. 7A).

Since Ubp6 is responsible for the recycling of ubiquitin molecules, the lack of Ubp6, coupled with an increased demand for ubiquitin because of the shortage of Rpl1, may lead to a shortage of ubiquitin [34]. If this is the case, then supplying additional ubiquitin might suppress the slow growth of the double mutant. Indeed, introduction into the double mutant of a 2 micron plasmid carrying a ubiquitin construct leads to growth comparable to the *rpl1bΔ* strain itself (Fig. 7B). It is interesting, however, that while supplemental ubiquitin leads to an increased growth rate of the double mutant during log phase, it does not cure the strain's inability to undergo a productive diauxic shift after the exhaustion of glucose (Fig. 7C). Note that the selective advantage of the supplemental ubiquitin maintains the plasmid in YPD medium.

Another protein identified in the SGA screen, Doa1, also has a role in maintaining ubiquitin levels in the cell [35] and in addition has been implicated in the vacuolar degradation of ribosomal subunits, termed ‘ribophagy’ [36]. Nevertheless, polysome analysis of the *doa1Δ rpl1bΔ* strain (T_1/2_ = 232±16 min) was more like wild type, showing little if any accumulation of Rpl1-deficient subunits, as estimated from the Rpl1/Rpl5 ratio determined by Western analysis (Fig. S2B). Thus, our data suggest that Doa1 and the ribophagy process are not responsible for the degradation of Rpl1-deficient subunits.

Although they had not been identified in the SGA screen, we studied the E3 ubiquitin ligase complex member, Rtt101, and its partner, Mms1, because they have been implicated in the degradation of aberrant 60S ribosomal subunits [37]. However, we found that deletion of neither gene had any effect on the turnover of RNA as seen in Fig. S3 (compare to Fig. 6C). This result suggests that there are multiple systems for eliminating aberrant ribosomal subunits.

### The inhibition of proteasome function elicits a stress response

**Figure 8:**
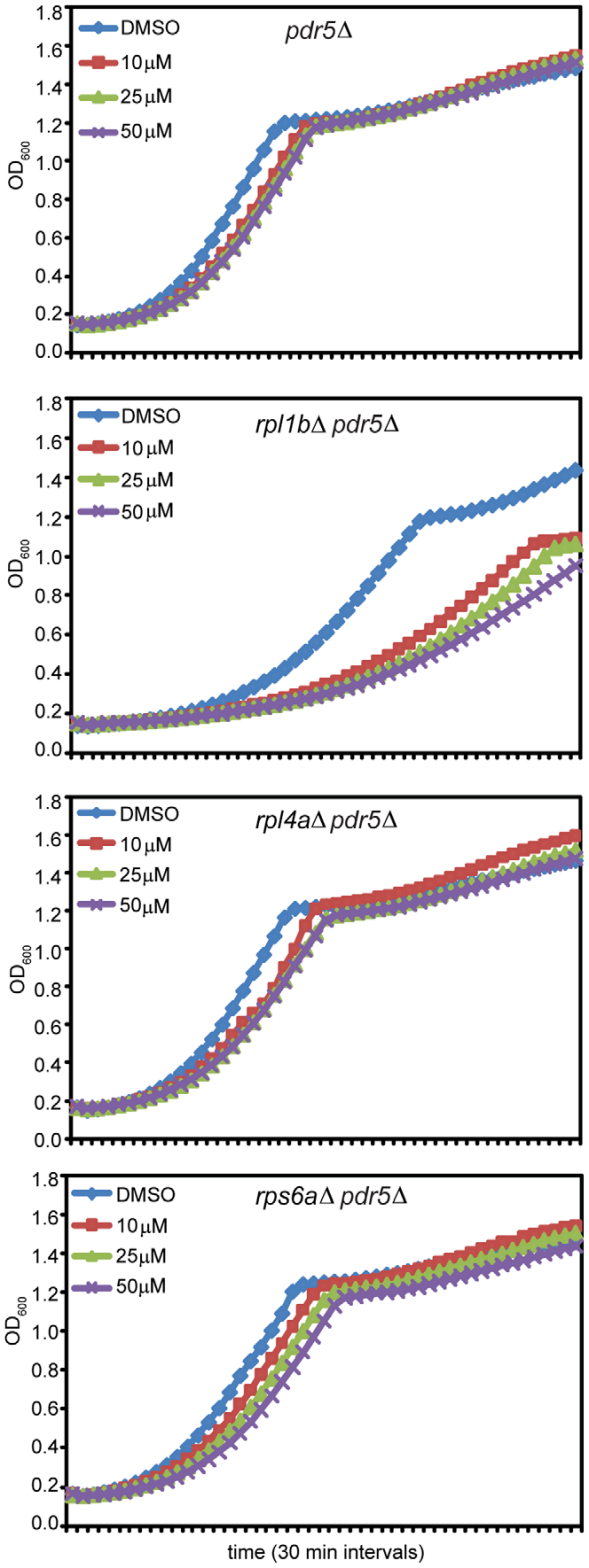
Growth of *rpl1b*Δ is highly sensitive to proteasome inhibition. Growth curves were recorded at 30°C for (A) *pdr5*Δ (wt), (B) *rpl1b*Δ *pdr5*Δ, (C) *rpl4a*Δ *pdr5*Δ, and (D) *rps6a*Δ *pdr5*Δ. Note that all strains used for MG132 experiments carry a deletion of *PDR5* to prevent drug efflux [38]. Strains were grown to log phase and inoculated to a final OD_600_ of ∼0.1 in YPD supplemented with 0.1% DMSO alone or with MG132 at concentrations of 10, 25, or 50 µM. Triplicate wells were loaded for each strain/drug treatment. Representative curves are shown. See Table 1 for compiled doubling times from all biological replicates.

The observation that a number of genes in the ubiquitin-proteasome complex were synthetic sick with *rpl1bΔ* led us to ask whether lack of proteasome function itself sensitizes cells haploinsufficient for Rpl1. To do so we employed the proteasome inhibitor MG132, using cells with a deletion of *PDR5* to prevent efflux of the drug [38]. The effects on growth were analyzed in the Bioscreen by inoculating log phase cells at an OD_600_ of ∼0.1 into growth wells containing DMSO or with MG132 at different concentrations. The resulting growth curves (Fig. 8) show clearly that the effect of even the lowest concentration of MG132 on growth of Δ*rpl1b* strain is far more drastic than on either the wildtype or the Δ*rpl4a* or Δ*rps6b* strains. The doubling times are shown in Table 1. The chemical-genetic interaction seen with MG132 serves to corroborate the genetic interactions of *rpl1bΔ* with the many members of the proteasome complex. It is interesting that the presence of MG132 seems to have a continuous effect during the ten hours of the growth curve in spite of the suggestion that it may be degraded within a few hours [39].

**Table 1:** Doubling times for strains following MG132 treatment.

Strain	MG132 conc. (µM)	Mean doubling time (min)	% increase from 0 µM
Y7092	0	97.8±2.4	0
	10	109.0±2.5	11.5
	25	114.8±3.8	17.4
	50	118.5±4.9	21.2
*rpl1b*Δ	0	180.7±4.6	0
	10	257.7±14.4	42.6
	25	272.7±7.5	50.9
	50	293.2±10.2	62.2
*rpl4a*Δ	0	109.7±2.7	0
	10	125.2±2.4	14.1
	25	131.0±2.2	19.4
	50	136.2±2.3	24.1
*rps6a*Δ	0	118.0±4.8	0
	10	136.9±6.3	16.0
	25	142.7±7.2	20.9
	50	148.8±9.6	26.1

Doubling times were calculated as in Materials and Methods, based on growth curves from the Bioscreen C™ reader as in Figure 8. Values shown are doubling times in minutes ± SE (n = 3).

**Figure 9:**
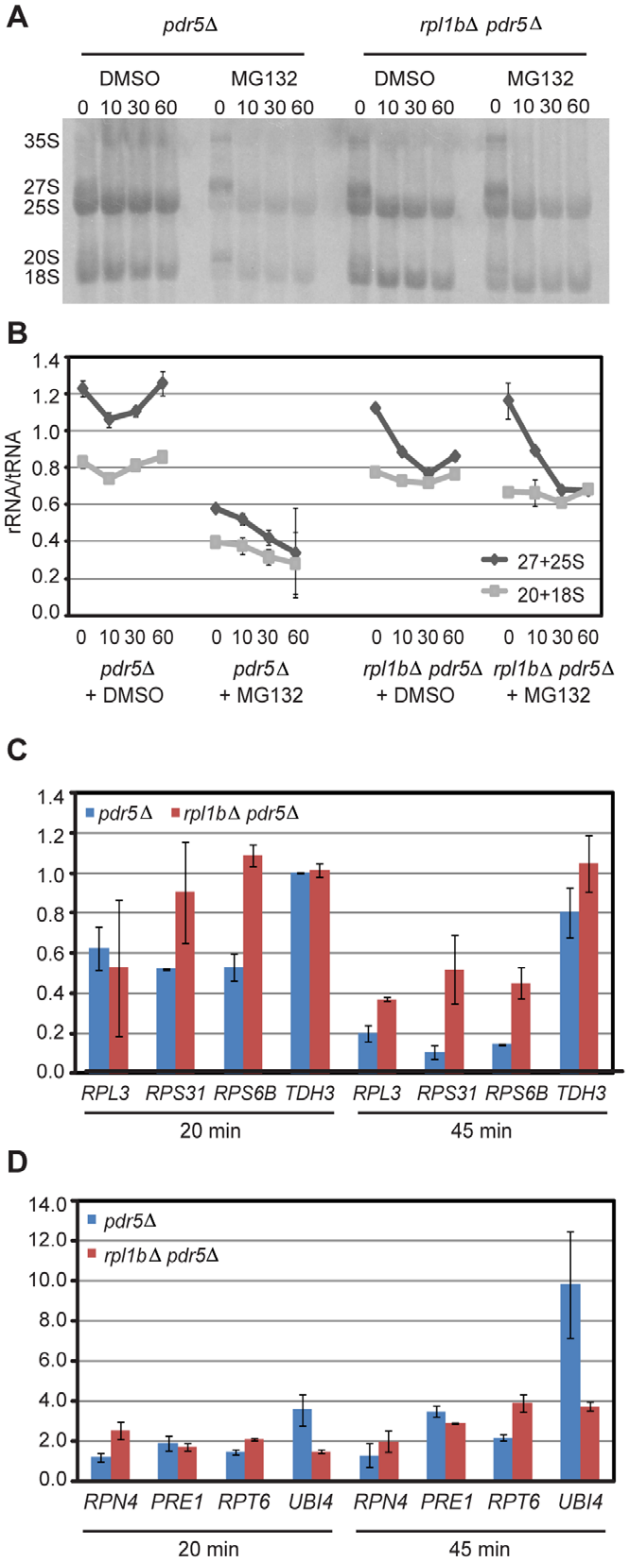
MG132 severely disrupts, and *Δrpl1b* partly restores ribosome synthesis. (A) A 15 minute pulse-chase with [C^3^H_3_]-methionine as in Fig. 6B. As in Fig. 8, these strains carry a deletion of *PDR5*. (B) Quantitative data from (A) where the CPM for the rRNA species is normalized to tRNA on the same gel. (C) qPCR determination of mRNA levels of RP genes and *TDH3* after MG132 treatment. C_T_ values were first normalized to *ACT1*. The values shown represent the fold change of mRNA level in MG132-treated cells relative to those treated with the DMSO solvent. (D) As in (C) for non-RP genes. Note the difference in scale.

Is the susceptibility of the Δ*rpl1b* strain to MG132 due to a failure to degrade aberrant cytoplasmic 60S subunits? A pulse-chase with [C^3^H_3_]-methionine, similar to that in Fig. 6B, gave a surprising result. Although the growth curves of Fig. 8 showed that wildtype cells were resistant to MG132, while Δ*rpl1b* cells were sensitive, pulse labeling showed just the opposite (Fig. 9A). After 30 minutes in MG132, both transcription and processing of rRNA in the wildtype strain was severely inhibited, while they continued at a substantial rate in the Δ*rpl1b* strain. Nevertheless, the degradation of 25S rRNA seen in the Δ*rpl1b* strain was still evident (Fig. 9B), proving that such degradation is not a function of the proteasome, directly or indirectly.

The reason for the repression of rRNA transcription and processing became clear when mRNA was analyzed. As with the rRNA, in wildtype cells RP mRNAs decline rapidly through the first 45 minutes of MG132 treatment, while in *rpl1bΔ* cells the RP mRNAs are less affected (Fig. 9C). Over a longer time, the RP mRNA levels recover (Fig. S4) as would be predicted from the mild influence of MG132 on the growth of wt cells (Fig. 8A). By contrast, the mRNA level for *RPN4*, encoding the transcription factor active at many proteasomal genes, rapidly increases, with concomitant increases in the mRNA levels of proteasomal genes (*RPT6* & *PRE1*) and a dramatic increase in a source for ubiquitin, *UBI4* (Fig. 9D). Thus, the extreme sensitivity of *rpl1b*Δ to MG132 is clearly not due to an inability to recover proteasome levels in the presence of the drug. A particularly intriguing contrast is the rapid decline in mRNA from *RPS31* (also known as *UBI3* since it encodes a ubiquitin-Rps31 fusion protein [40]) vs. the strong induction of *UBI4*, encoding four ubiquitins in tandem.

Thus, as has been reported for mammalian cells [41], MG132 initially represses ribosome synthesis. It seems likely that in *S. cerevisiae* this repression of ribosome synthesis mimics the stress response seen when the ER is insulted by drugs or mutation [42], presumably due to a failure to deal with unfolded proteins in the ER. We have shown that this stress response is partially ablated by the haploinsufficiency of Rpl1 [43], and we suggest that the data of Fig. 9 is another manifestation of that effect. Presumably the failure to assemble ribosomes correctly provides a signal that partially overrides the repression of ribosome synthesis due to ER stress.

## Discussion

Ribosomal protein L1 is unusual in a number of aspects. It occupies a prominent site on the large ribosomal subunit, forming a lateral protuberance associated with a single loop of the 25S rRNA. During the course of a translation step it moves more than any other RP, arcing between two quite distinct orientations in connection with movement of uncharged tRNA from the P-site to the E-site, and then again to facilitate the exit of tRNA from the E-site [16]–[18]. While these functions appear to be conserved from prokaryotes to mammals, Rpl1 is essential for growth of *S. cerevisiae* [21] but not for *E. coli* [44], where its absence reduces the growth rate by only 50% [18]. It thus seems surprising that haploinsufficiency for Rpl1 should have such an effect on growth. We suggest that this is may be due to the fact that Rpl1 is an unusual eukaryotic RP in that 60S ribosomes lacking Rpl1 can be exported from the nucleus to the cytoplasm (Fig. 3B) and can even be found on polysomes (Fig. 6).

Although yeast cells cannot grow without Rpl1 that does not necessarily imply that translation cannot occur on ribosomes lacking Rpl1, albeit at a reduced rate. What is clear is that 60S subunits lacking Rpl1 compete poorly for association with initiating 43S complexes on mRNA (Fig. 6). The role of Rpl1 in the translation process, namely the moving of an empty tRNA into and out of the E site, could impact translation in several ways. On the one hand, lack of Rpl1 could simply slow up each step, since the *E. coli* data suggest that Rpl1 is not essential for the process. On the other hand, some models of translation postulate that filling of the A site with a new aa-tRNA is coupled to the exit of tRNA from the E site [29]. If so, then lack of Rpl1 could affect the accuracy of selection of the A site aa-tRNA. Although we were unable to demonstrate such miscoding directly, it may occur with enough frequency that the ubiquitin-proteasome system is called upon to degrade an unusual flux of denatured proteins. It is interesting that although a number of mutant RP genes have been identified as a cause of human disease, there are as yet no examples of human SNPs that alter the translation of the human Rpl1, Rpl10a. It may be that haploinsufficiency for this gene is lethal.

One reason for our selection of Rpl1 to study is that we had previously found that a nonsense mutation in *RPL1B* suppressed one element of the ER stress response, namely the repression of ribosome synthesis, expressed through repression of both Pol I transcription of rRNA genes and Pol II transcription of RP genes [43]. It was therefore interesting to find that loss of *RPL1B* also partially suppresses the temporary repression of ribosome synthesis brought about by inhibition of the proteasome with MG132 (Fig. 9). It remains to be seen whether this is due simply to a deficiency of 60S subunits, to the effects of mistranslation by ribosomes lacking Rpl1, or to a shortage of ubiquitin.

One element of cell physiology that has become clearer from our results is the yin-yang relationship of the ribosome synthesis complex and the proteasome complex. Whereas nearly any stress leads to rapid repression of ribosome synthesis [1], such stress leads to induction of proteasome synthesis [45]. The most vibrant example is the contrast between *RPS31* (*UBI3*) and *UBI4* (Fig. 9C). During normal growth the production of ubiquitin derives from three ubiquitin-RP fusion genes, *RPL40A*&*B* and *RPS31*. Thus, it matches the production of ribosomes, and of translation. By contrast, under stress ubiquitin can be produced in large amounts even as ribosome synthesis is repressed.

The machinery that detects and destroys aberrant ribosomes remains unknown. While there is some evidence that Rtt101 and Mms1 play such a role for aberrant 60S subunits, our experiments showed that neither of these had an effect on the turnover of 60S subunits lacking Rpl1 (Figs. 6, S3). Nor, apparently, does the proteasome (Fig. 9B). There clearly remain surveillance mechanisms that have yet to be identified.

An interesting element that remains unexplained is the fact that only the strain carrying the double deletion *ubp6*Δ *rpl1b*Δ appears to be functionally petite, in that it does not undergo the diauxic shift (Fig. 7B) and will not grow on plates whose only C source is glycerol. While provision of extra ubiquitin suppresses the slow growth phenotype of this double mutant, it does not suppress this failure of oxidative growth. The role in mitochondrial biosynthesis and function that is played by the cytoplasmic ribosome and cytoplasmic translation is fruitful for further study.

## Materials and Methods

### Strains

Yeast strains used in this study are listed in Table S1. pUB175 is a 2 micron, *URA3* plasmid carrying ubiquitin driven by the *CUP1* promoter.

### Growth analysis

Growth at 30°C was measured using a Bioscreen C® Microbiology Reader (Growth Curves USA), which recorded OD_600_ readings from 100-well plates every 30 minutes for up to 72 hours. Strains were diluted in growth medium from single colonies or log-phase cultures prior to inoculation, in triplicate, of 150 µl media per well in Bioscreen plates. To prevent clumping of cells, we added Nonidet-P40 to minimal (dropout) media at a final concentration of 0.2%. For MG132 sensitivity experiments, strains were grown to log phase and inoculated into YPD +/− drug at a final OD_600_ of ∼0.1. Doubling times were calculated based on the time for each strain to grow from OD_600_ 0.1 to 0.4.

### Polysome analysis

Strains were grown to log phase (OD_600_ ∼0.8–1.0). Cycloheximide was added to a final concentration of 100 µg/ml and cells were chilled immediately on ice prior to collection. Lysis was carried out in LHB buffer (0.1 M NaCl, 0.03 M MgCl_2_, 0.01 M Tris pH 7.4, 100 µg/ml cycloheximide, 200 µg/ml heparin) with an equal volume of glass beads in a BeadBeater8, clarified by centrifugation at 14,000 g for 15 min, and the supernatant layered on 11 ml gradients. Sucrose density gradients were prepared with the appropriate amount of sucrose [10–50% (w/v) or 5–20% (w/v)] in TMN buffer (0.05 M Tris-Ac pH 7.0, 0.05 M NH_4_Cl, 0.012 M MgCl_2_). For low magnesium gradients, LHB and TMN buffers were prepared using 1.5 mM MgCl_2_. Gradients were centrifuged for 2.5–4 h at 40K rpm in a SW41 rotor, A_260_ was read using an ISCO UA-5 absorbance detector, and 500 µl fractions were collected into 1/10^th^ volume of 20% SDS.

### Purification of cytoplasm extract

Preparation of cytoplasm extract was carried out essentially as described [46]. Cells were grown to log phase (OD_600_ ∼1.0), washed in water, and spheroplasts prepared by resuspending in 1/10^th^ volume 1 M sorbitol+1% Glusulase (PerkinElmer) and incubating for 1 h at room temperature. Spheroplasts were diluted into 10 volumes synthetic complete medium containing 0.4 M MgSO_4_ as osmotic support. After 2 h of gentle swirling at 30°C, the culture was treated with 100 µg/ml cycloheximide, chilled over frozen 1 M sorbitol, collected (2 min, 10K rpm), and washed with cold 1 M sorbitol. Spheroplasts were lysed at 4°C for 15 min in 1/10^th^ volume cell fractionation buffer (CFB, pH 6.5: 0.01 M NaCl, 0.01 M PIPES, 0.005 M MgCl_2_, 0.001 M DTT, 0.01% spermidine) and lysis completed (but leaving nuclear and mitochondrial membranes intact) by adding 0.1% saponin (British Drug Houses, Ltd.). Centrifugation at 12K rpm (∼12× *g*) for 10 min at 4°C yielded the cytoplasmic fraction. Note that this technique yields a very pure cytoplasmic fraction, but a nuclear fraction that is heavily contaminated with residual cytoplasm and semibroken cells.

### Western blotting

Equal volumes of polysome gradient fractions were run on 4–15% polyacrylamide gradient gels using a Tris-Glycine-SDS buffer system and were electro-blotted onto nitrocellulose membrane. Membranes were blocked in Odyssey Blocking Buffer (li-cor Biosciences) and probed overnight at 4°C with α-L1 (kind gift of François Lacroute) and α-L5 (kind gift of John Woolford) rabbit polyclonal antibodies at 1∶5000. Probing with IRDye680 Goat Anti-Rabbit secondary antibody (li-cor Biosciences) was followed by analysis using an Odyssey infrared imaging system.

### RNA extraction, Northern blotting, and RT-qPCR

Total RNA extraction and Northern blotting was performed essentially as described previously [47] from cell pellets resuspended in AE buffer (50 mM Na-Ac pH 5.3, 10 mM EDTA pH 7.0) using glass bead lysis and AE-saturated phenol extraction. For isolating RNA from sucrose gradient fractions, 10 µg glycogen (Sigma), 1/10 volume of 3 M NaAc pH 5.5, and 2.5 volumes 95% ethanol were added to each fraction and stored overnight at −20°C. The RNA was collected by centrifugation, washed once with 70% ethanol, and resuspended in 15 µl water.

For RT-qPCR, 1 µg of total RNA treated with RQ1 DNase (Promega) was used for reverse transcription using SuperScript III (Invitrogen) and both oligo(dT)_20_ and random hexamer primers. cDNAs were diluted 100-fold and qPCR was conducted using an Applied Biosystems ABI Prism 7900HT Fast Real-Time PCR system and ABsolute Blue QPCR SYBR Green ROX Mix (Thermo Scientific). Analysis was carried out using the comparative ΔΔC_T_ method. Primers used for qPCR are listed in Table S2.

### Labelling with [C^3^H_3_]-methionine and with ^32^P

Strains were labeled with [C^3^H_3_]-methionine and with ^32^P as described [48]. Briefly, log phase cells (OD_600_ ∼0.8–1.0) in methionine-free medium were labeled with 60 µCi/ml [C^3^H_3_]-methionine for 3 or 15 min at 30°C, followed by a chase of 1 mg/ml unlabeled methionine. Samples of 1.5–2 ml were poured over ice at intervals post-chase, collected via centrifugation and the pellet flash-frozen in liquid nitrogen. Total RNA was extracted and 5 µg per sample run on a 1.5% denaturing agarose gel and transferred to nylon membrane. Following UV crosslinking and drying, membranes were dipped twice in EN^3^HANCE (Perkin-Elmer), allowed to dry, and subjected to autoradiography (2–5 days at −80°C). In some cases, using the x-ray film as a guide, 25S rRNA, 18S rRNA, and tRNA bands were cut from the membrane and activity measured using a scintillation counter.

For incorporation of ^32^P, cultures were grown to log phase in medium depleted of inorganic PO_4_. After 60 min in medium with the appropriate C source, 100 µCi/ml ^32^PO_4_ was added. After 60 min more, cells were harvested and analyzed as described above, lysed in low Mg^2+^ LHB and the clarified lysate was analyzed as described above, except that the Mg^2+^ concentration was 1.5 mM in both LHB and TMN buffers.

## Supporting Information

Figure S1 **Nuclear-free cytoplasmic extract.** Extracts were prepared as in Materials and Methods, and equal volumes cytoplasmic (C) and nuclear (N) subcellular fractions were run on the same blot and hybridized with antibodies to histone H2B (nuclear marker) and Rpl3 (nuclear/cytoplasmic) to demonstrate that cytoplasmic fractions are nuclear-free. Different channels were used to detect the IR Dye secondary antibodies for H2B (700 channel) and Rpl3 (800 channel) on the same blot using Li-Cor.
(TIF)

Figure S2 **Polysome profiles and western blots for SGA interactors.** Polysome profiles of (A) *ubp6*Δ and *ubp6*Δ *rpl1b*Δ and (B) *doa1*Δ and *doa1*Δ *rpl1b*Δ; 8.4 (*ubp6*Δ *rpl1b*Δ) or 11 (all other strains) A_260_ units of whole cell lysate was layered onto 7–47% sucrose gradients and centrifuged for 2.5 h at 40K rpm. Top of the gradient is at left. Western blots of equal volumes of each gradient fraction, probed with α-Rpl1 and α-Rpl5 is shown below the polysome profiles for double KO strains.
(TIF)

Figure S3 **Mms1 and Rtt101 are not involved in degradation of subunits in** ***rpl1b*** **Δ.** Strains were grown to log phase and subjected to a 15 min [C^3^H_3_]-methionine pulse followed by a cold met chase. 5 µg total RNA from each sample was blotted and subjected to autoradiography, and bands corresponding to 27S+25S and 20S+18S rRNA were cut out and counted directly using a scintillation counter as in Fig. 6. Graph shows 25S/18S ratio ± SD (n = 2).
(TIF)

Figure S4 **Repression and recovery of RP mRNAs following MG132 treatment.** (A) qPCR determination of mRNA levels of RP genes following up to 180 minutes of incubation with MG132. C_T_ values were first normalized to *ACT1*. The values shown represent the fold change of mRNA level in MG132-treated cells relative to those treated with the DMSO solvent. (B) As in (A) for non-RP genes. Note the difference in scale.
(TIF)

Table S1 Strains used in this study.
(DOC)

Table S2 Primers used in qPCR.
(DOC)

Table S3 Total RNA content of RP and proteasome mutants compared to wildtype. Micrograms of total RNA per OD_600_ unit of cells were determined following RNA extraction for an equal volume of cells for each strain. Values shown are normalized to wildtype.
(DOC)

Table S4 rRNA species in RP and proteasome mutants compared to wildtype. Relative amounts of different rRNA species were determined by Northern blot quantitation using Phosphorimager software.
(DOC)
