# Supplementary figures and images for: Eukaryotic Cells Producing Ribosomes Deficient in Rpl1 Are Hypersensitive to Defects in the Ubiquitin-Proteasome System
Source: PLoS One. 2011 Aug 12;6(8):e23579. doi: 10.1371/journal.pone.0023579 (PMC3155557; doi:10.1371/journal.pone.0023579)

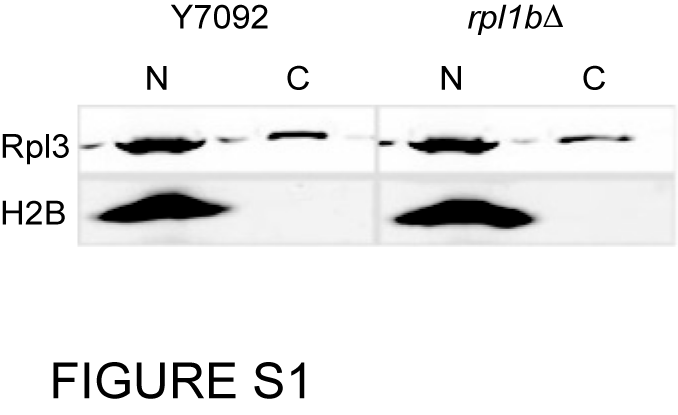

Supplement: Figure S1 — Nuclear-free cytoplasmic extract. Extracts were prepared as in Materials and Methods, and equal volumes cytoplasmic (C) and nuclear (N) subcellular fractions were run on the same blot and hybridized with antibodies to histone H2B (nuclear marker) and Rpl3 (nuclear/cytoplasmic) to demonstrate that cytoplasmic fractions are nuclear-free. Different channels were used to detect the IR Dye secondary antibodies for H2B (700 channel) and Rpl3 (800 channel) on the same blot using Li-Cor. (TIF) [file pone.0023579.s001.tif]

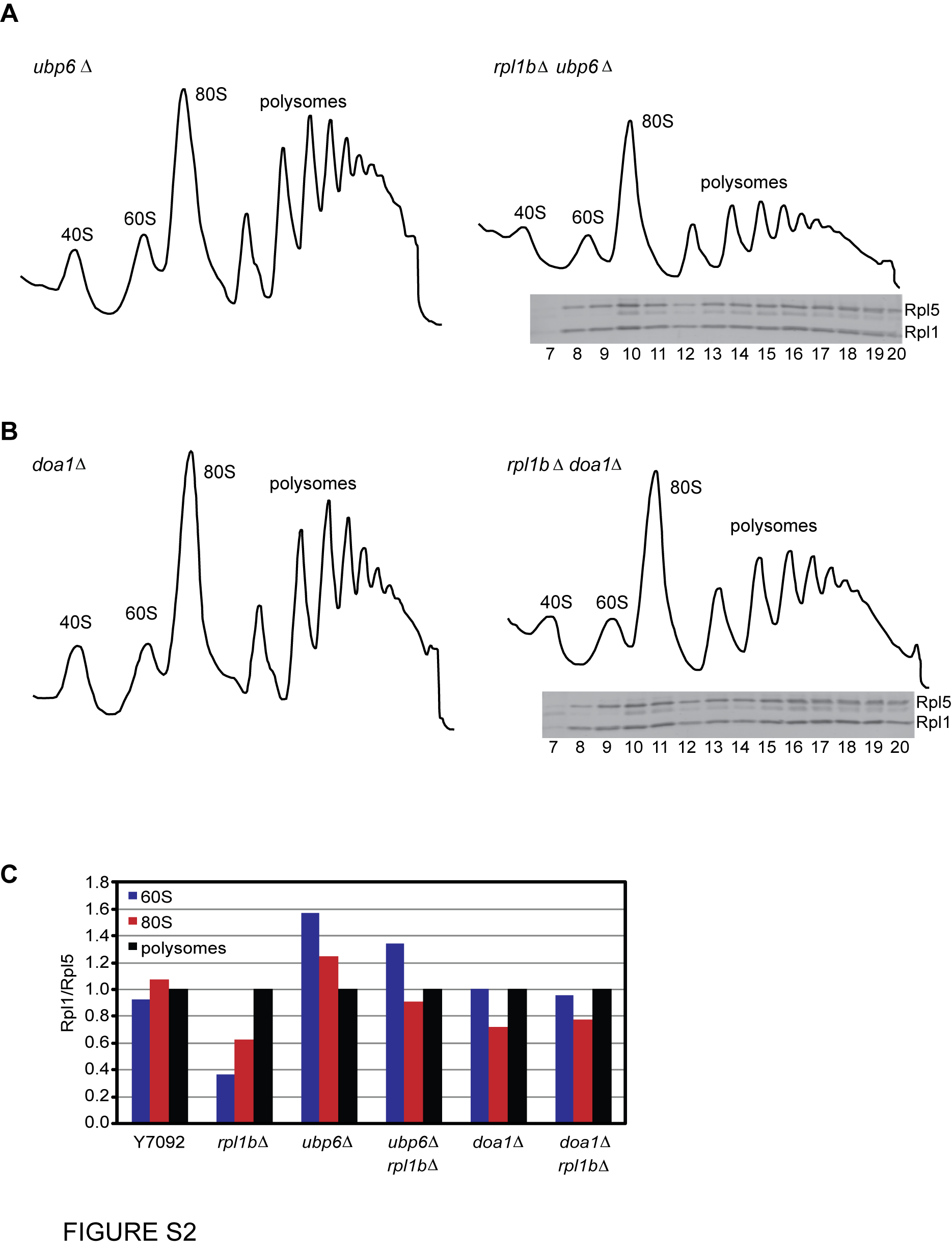

Supplement: Figure S2 — Polysome profiles and western blots for SGA interactors. Polysome profiles of (A) ubp6Δ and ubp6Δ rpl1bΔ and (B) doa1Δ and doa1Δ rpl1bΔ; 8.4 (ubp6Δ rpl1bΔ) or 11 (all other strains) A260 units of whole cell lysate was layered onto 7–47% sucrose gradients and centrifuged for 2.5 h at 40K rpm. Top of the gradient is at left. Western blots of equal volumes of each gradient fraction, probed with α-Rpl1 and α-Rpl5 is shown below the polysome profiles for double KO strains. (TIF) [file pone.0023579.s002.tif]

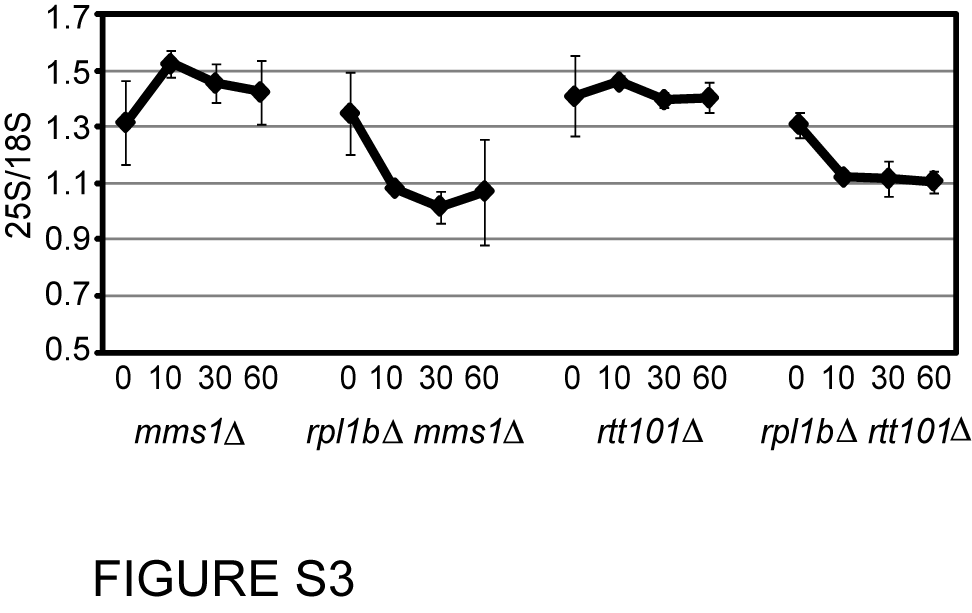

Supplement: Figure S3 — Mms1 and Rtt101 are not involved in degradation of subunits in rpl1b Δ. Strains were grown to log phase and subjected to a 15 min [C3H3]-methionine pulse followed by a cold met chase. 5 µg total RNA from each sample was blotted and subjected to autoradiography, and bands corresponding to 27S+25S and 20S+18S rRNA were cut out and counted directly using a scintillation counter as in Fig. 6. Graph shows 25S/18S ratio ± SD (n = 2). (TIF) [file pone.0023579.s003.tif]

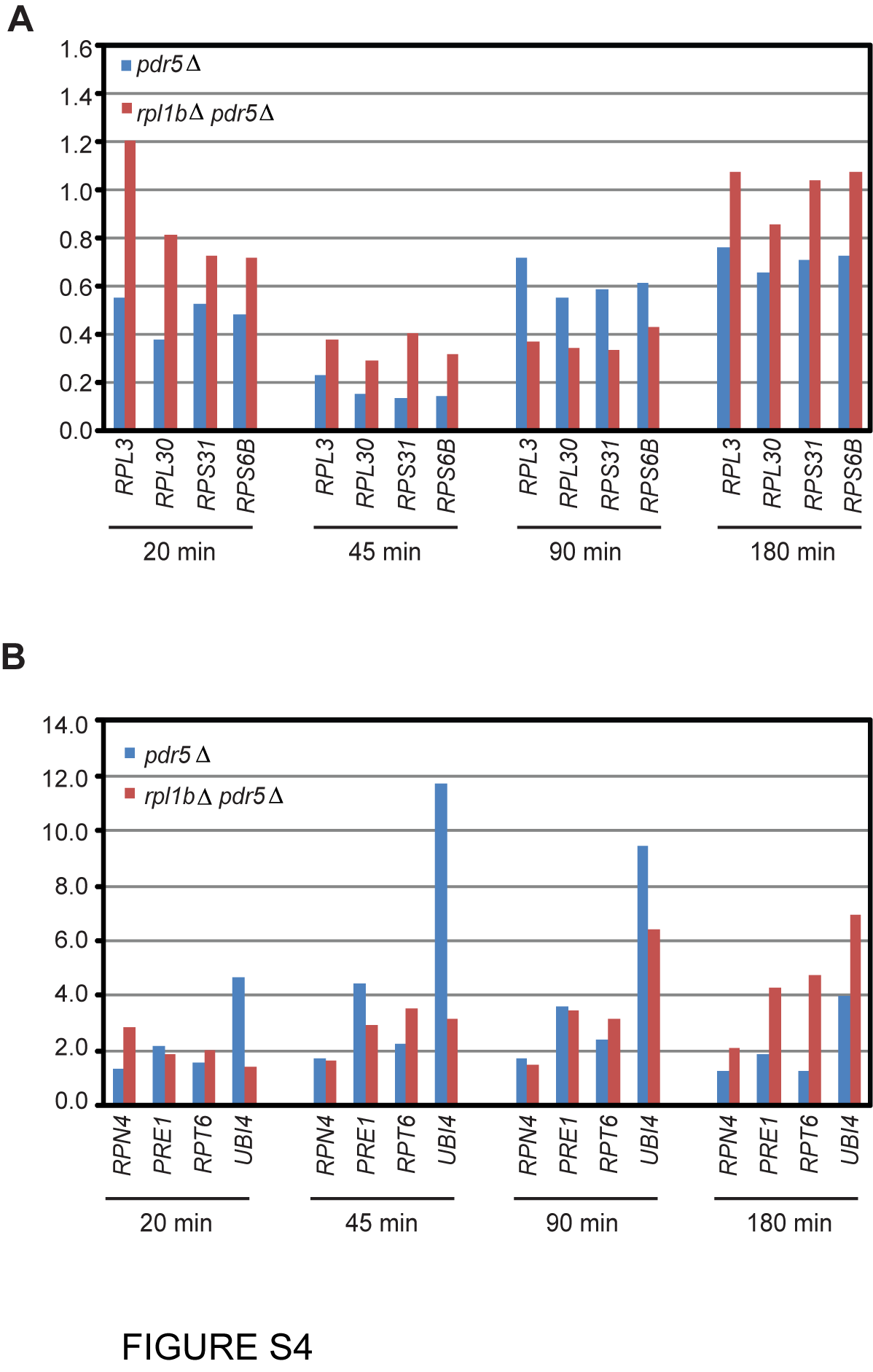

Supplement: Figure S4 — Repression and recovery of RP mRNAs following MG132 treatment. (A) qPCR determination of mRNA levels of RP genes following up to 180 minutes of incubation with MG132. CT values were first normalized to ACT1. The values shown represent the fold change of mRNA level in MG132-treated cells relative to those treated with the DMSO solvent. (B) As in (A) for non-RP genes. Note the difference in scale. (TIF) [file pone.0023579.s004.tif]
