# Supplementary material for: Eukaryotic Cells Producing Ribosomes Deficient in Rpl1 Are Hypersensitive to Defects in the Ubiquitin-Proteasome System
Source: PLoS One. 2011 Aug 12;6(8):e23579. doi: 10.1371/journal.pone.0023579 (PMC3155557; doi:10.1371/journal.pone.0023579)
Supplement: Table S1 — Strains used in this study. (DOC) [file pone.0023579.s005.doc]

Table S1. Strains used in this study.

| **Strain** | **Genotype** | **Reference/Source** |
| --- | --- | --- |
| Y7092 | MATα *can1*Δ::STE2pr-Sp_*his5* *lyp1*Δ *his31*Δ *leu2*Δ*0* *ura3*Δ*0* *met17*Δ*0 LYS2+* | Tong and Boone |
| BY4741 | MAT**a** *his3Δ1* *leu2Δ0* *met15Δ0* *ura3Δ0* | Open Biosystems |
| KBM18 | Y7092**^+^** *rpl1a*Δ::*nat^R^* | This study |
| KBM23 | Y7092**^+^** *rpl1b*Δ::*nat^R^* | This study |
| KBM22 | Y7092**^+^** *rpl4a*Δ::*nat^R^* | This study |
| KBM17 | Y7092**^+^** *rpl4b*Δ::*nat^R^* | This study |
| KBM24 | Y7092**^+^** *rps6a*Δ::*nat^R^* | This study |
| KBM13 | Y7092**^+^** *MET17* | This study |
| KBM14 | Y7092**^+^** *MET17* *rpl1b*Δ::*nat^R^* | This study |
| KBM15 | Y7092**^+^** *MET17* *rpl4a*Δ::*nat^R^* | This study |
| KBM25 | Y7092**^+^** *MET17* *rps6a*Δ::*nat^R^* | This study |
| KBM20 | Y7092**^+^** *MET17* *rpl1b*Δ::*nat^R^ G418^R^::GAL1::RPL1A* | This study |
| KBM21 | Y7092**^+^** *MET17* *rpl4a*Δ::*nat^R^ G418^R^::GAL1::RPL4B* | This study |
| KBM55 | Y7092**^+^** *MET17* *rps6a*Δ::*nat^R^ G418^R^::GAL1::RPS6B* | This study |
| JO241 | BY4741**^+^** *ubp6*Δ::*G418^R^* | This study |
| JO217 | BY4741**^+^** *doa1*Δ::*G418^R^* | This study |
| KBM32 | Y7092**^+^** *rpl1b*Δ::*nat^R^* *ubp6*Δ::*G418^R^* | This study |
| KBM31 | Y7092**^+^** *rpl1b*Δ::*nat^R^* *doa1*Δ::*G418^R^* | This study |
| KBM68 | Y7092**^+^** *MET17* *mms1*Δ::*G418^R^* | This study |
| KBM69 | Y7092**^+^** *MET17* *rtt101*Δ::*G418^R^* | This study |
| KBM70 | Y7092**^+^** *MET17* *rpl1b*Δ::*nat^R^ mms1*Δ::*G418^R^* | This study |
| KBM71 | Y7092**^+^** *MET17* *rpl1b*Δ::*nat^R^ rtt101*Δ::*G418^R^* | This study |
| KBM53 | Y7092**^+^** *MET17* *pdr5*Δ::*G418^R^* | This study |
| KBM54 | Y7092**^+^** *MET17* *rpl1b*Δ::*nat^R^ pdr5*Δ::*G418^R^* | This study |
| KBM58 | Y7092**^+^** *MET17* *rpl4a*Δ::*nat^R^ pdr5*Δ::*G418^R^* | This study |
| KBM59 | Y7092**^+^** *MET17* *rps6a*Δ::*nat^R^ pdr5*Δ::*G418^R^* | This study |

* nat^R^ = nourseothricin resistance marker
