# Supplementary material for: Eukaryotic Cells Producing Ribosomes Deficient in Rpl1 Are Hypersensitive to Defects in the Ubiquitin-Proteasome System
Source: PLoS One. 2011 Aug 12;6(8):e23579. doi: 10.1371/journal.pone.0023579 (PMC3155557; doi:10.1371/journal.pone.0023579)
Supplement: Table S2 — Primers used in qPCR. (DOC) [file pone.0023579.s006.doc]

**Table S2. Primers used in qPCR.**

| **Oligo Name** | **Gene** | **Oligo Sequence (5' - 3')** |
| --- | --- | --- |
| JW2748 | ACT1 | GTA CCA CCA TGT TCC CAG GT |
| JW2749 | ACT1 | CCA CCA ATC CAG ACG GAG TA |
| JW2752 | TDH3 | GGT TGT TAA GGC TGC CGC TG |
| JW2753 | TDH3 | AGC GGA AGC ATC GAA GAT GG |
| JW2754 | RPL1A | CTG GCA AAA TGT TGG TTC CTT G |
| JW2755 | RPL1A | CTG AAA GCG TTA AAC TAT GAA AAC ACG |
| JW2756 | RPL1B | AGA ACT GGC AAA ATG TTG GTT CC |
| JW2757 | RPL1B | GAT GGA GAT ATG TAT TAA TCG CTC GGA |
| JW2760 | RPL4B | TTA CGC CAA GGT CTT TGC TGC |
| JW2761 | RPL4B | GTC ATG CTT CAA GGT TTC GGC |
| JW2860 | U1 | CGC GGA AGG CGT GTT TGC TG |
| JW2861 | U1 | GGC AGA AGA AAC AAA GGG CCC CA |
| JW2864 | U2 | GCA ATG GAA GGC GTT TGC TGG G |
| JW2865 | U2 | GCC GCC GGC ATT CTT CAA ATC C |
| JW2868 | RPL4A | CCC AAG AAC AAT CAT CGA AAT GTC CCG |
| JW2869 | RPL4A | ACA GCT GGC AAT GGC AAG GCA |
| JW2872 | RPL3 | GCT GTC ACC AAG GGT CAC GGT |
| JW2873 | RPL3 | CGT GGG CTG GAT GCC AAG CA |
| JW2876 | RPL30 | TCA TCA TTG CCG CTA ACA CTC CAG |
| JW2877 | RPL30 | ACC GAC AGC AGT ACC CAA TTC GT |
| JW2892 | RPS6B | AGA GAA GCT GCT GCC GAA TAC GC |
| JW2893 | RPS6B | AGC TCT TCT CTT TCT GAT TTC GGC CT |
| JW2904 | RPS31-Ubi | AAC CCA ACT TGT GGT GCT GGT GT |
| JW2905 | RPS31-Ubi | AGC GTT AAC CTT GTA GAC GGA ATG ACA |
| JW2920 | UBI4 | AGA AGA TGG TAG AAC GCT GTC GGA C |
| JW2921 | UBI4 | GCG AGG ACT GAT CAG TTA CCA CCC C |
| JW2922 | RPN4 | CGA CTG CAT CAC CAT CGG CCC |
| JW2923 | RPN4 | TGG GCA CCA CAC GGT TCA TTT GT |
| JW2924 | PRE1 | GGT GAC ACC GTT CAA TTC GCC G |
| JW2925 | PRE1 | AGA TAC TGC TTG TGG AGA GAG CTC A |
| JW2926 | RPT6 | CCT CCA AGT GTC GCA GCT AGA GC |
| JW2927 | RPT6 | TCG GCA CCA GAA CAA CCG TTC A |
| JW2932 | RPL1A&B | CCC AAC CCC AGT TTC TCA CAA CGA |
| JW2933 | RPL1A&B | ACG TTA CCA ACG GCA ACA GCC A |
| JW2934 | RPL4A&B | GCT GGC CAA GCT ACT CAA AAG CG |
| JW2935 | RPL4A&B | TCA GCA GCA AAG ACC TTG GCG T |
