# Supplementary material for: Eukaryotic Cells Producing Ribosomes Deficient in Rpl1 Are Hypersensitive to Defects in the Ubiquitin-Proteasome System
Source: PLoS One. 2011 Aug 12;6(8):e23579. doi: 10.1371/journal.pone.0023579 (PMC3155557; doi:10.1371/journal.pone.0023579)
Supplement: Table S3 — Total RNA content of RP and proteasome mutants compared to wildtype. Micrograms of total RNA per OD600 unit of cells were determined following RNA extraction for an equal volume of cells for each strain. Values shown are normalized to wildtype. (DOC) [file pone.0023579.s007.doc]

Table S3. Total RNA content of RP and proteasome mutants compared to wildtype.

| **Strain** | **RNA per OD_600_, vs. wt** |
| --- | --- |
| Y7092 | 1.0 |
| *rpl1b*Δ | 0.7 |
| *ubp6*Δ | 0.8 |
| *rpl1b*Δ *ubp6*Δ | 0.5 |
| *doa1*Δ | 0.9 |
| *rpl1b*Δ *doa1*Δ | 0.6 |
