# Supplementary material for: Eukaryotic Cells Producing Ribosomes Deficient in Rpl1 Are Hypersensitive to Defects in the Ubiquitin-Proteasome System
Source: PLoS One. 2011 Aug 12;6(8):e23579. doi: 10.1371/journal.pone.0023579 (PMC3155557; doi:10.1371/journal.pone.0023579)
Supplement: Table S4 — rRNA species in RP and proteasome mutants compared to wildtype. Relative amounts of different rRNA species were determined by Northern blot quantitation using Phosphorimager software. (DOC) [file pone.0023579.s008.doc]

**Table S4. rRNA species in RP and proteasome mutants compared to wildtype.**

| **Strain** | **rRNA species, vs. wildtype** | | | | |
| --- | --- | --- | --- | --- | --- |
|  | **35S** | **27S** | **20S** | **25S** | **18S** |
| **Y7092** | 1.00 | 1.00 | 1.00 | 1.00 | 1.00 |
| ***rpl1b*Δ** | 0.99 | 0.87 | 0.92 | 1.09 | 1.11 |
| ***ubp6*Δ** | 0.62 | 0.98 | 1.56 | 1.09 | 0.75 |
| ***rpl1b*Δ *ubp6*Δ** | 1.23 | 0.89 | 1.03 | 0.87 | 0.49 |
| ***doa1*Δ** | 0.07 | 0.27 | 0.46 | 0.51 | 0.49 |
| ***rpl1b*Δ *doa1*Δ** | 0.44 | 0.37 | 0.41 | 0.39 | 0.35 |

|  |  |  |  |  |
| --- | --- | --- | --- | --- |
|  |  |  |  |  |
|  |  |  |  |  |
|  |  |  |  |  |
|  |  |  |  |  |
|  |  |  |  |  |
